# Septorhinoplasty among Patients who Visited the Outpatient Department of Otolaryngology-Head and Neck Surgery of a Tertiary Care Center: A Descriptive Cross-sectional Study

**DOI:** 10.31729/jnma.6503

**Published:** 2021-05-31

**Authors:** Nain Bahadur Mahato, Deepak Regmi, Meera Bista

**Affiliations:** 1 Department of Otolaryngology-Head and Neck Surgery, Kathmandu Medical College and Teaching Hospital, Sinamangal, Kathmandu, Nepal

**Keywords:** *aesthetic*, *prevalence*, *satisfaction*, *septorhinoplasty*

## Abstract

**Introduction:**

The nose is the central most part of the face, so any nasal deformities may cause a high level of psychological distress on one's quality of life. Septorhinoplasty is a surgical procedure used to correct both functional as well as aesthetic problems of the nose. It enhances facial harmony and increases self-confidence. The objective of this study is to find out the prevalence of out-patients who underwent septorhinoplasty in a tertiary care centre in Nepal.

**Methods:**

A descriptive cross-sectional study among 5,760 patients who visited the outpatient department of Otolaryngology-Head and Neck Surgery of Kathmandu Medical College from April 2019 to September 2020. Ethical approval was taken from the Institutional Committee of Kathmandu Medical College. A convenient sampling technique was used. The preoperative and postoperative assessment was done with the help of a rhinoplasty outcome evaluation questionnaire. Statistical Package for the Social Sciences is used for analysis. Point estimate at 95% Confidence Interval was calculated along with frequency and proportion for binary data.

**Results:**

The prevalence of septorhinoplasty was 117 (2.03%) during the study period (95% Confidence Interval= 1.66-2.40). Among 117 patients, 67 (57.3%) were males and 50 (42.7%) were females. Among 117 patients, 85 (72.6%) patients underwent an open approach, while 32 (27.4%) patients underwent closed approach rhinoplasty for the correction of both aesthetic and functional problems.

**Conclusions:**

This study concludes the prevalence of septorhinoplasty is low which may be due to the COVID-19 pandemic during the study period.

## INTRODUCTION

Septorhinoplasty is the surgical procedure used to correct breathing problems related to the nose (septoplasty) or correct disfigurement resulting from trauma or birth defects (rhinoplasty). Rhinoplasty enhances the facial harmony and proportions of the nose which affects self-esteem that imparts a psychological benefit. Most of the rhinoplasty studies reported high rates of satisfaction and enhanced social confidence.^1,2^

In the last decades, there has been an increasing interest in the assessment of results of surgical procedures in several medical specialities.^3^ Concerning facial plastic surgery, patient satisfaction studies represent a poorly developed area, with validated instruments for objective and subjective assessment of results being rarely available.^4-6^

The main objective of this study is to find out the prevalence of patients who have undergone septorhinoplasty among patients who visited the outpatient department of Otolaryngology-Head and Neck Surgery of a tertiary care centre in Nepal.

## METHODS

This is a descriptive cross-sectional study among 5,760 patients who visited the outpatient department of Otolaryngology-Head and Neck Surgery of Kathmandu Medical College from April 2019 to September 2020. Ethical clearance was taken from the ethical clearance committee of KMCTH. Patients who gave consent for the study were included in the study, and those who did not give consent were excluded. The convenient sampling technique was used, and the sample size was calculated using the formula,


$$\begin{array}{ll} \text{n} & \text{= }{\text{Z}}^{\text{2}}\times \text{p}\times \text{q}/{\text{e}}^{\text{2}}\\ & \text{= }{\left(1.96\right)}^{\text{2}}\times 0.5\times (1-0.5)/{(\text{0.02)}}^{\text{2}}\\ & \text{= }2401 \end{array}$$

Where,

n = minimum required sample size
Z = 1.96 at 95% Confidence Interval (CI)
p = prevalence taken as 50% for maximum sample size
q = 1-p
e = margin of error, 2%

Since, the convenient sampling method was done, by doubling the sample size, we get, 4802. However, the total sample taken was 5,760.

Detailed history, clinical examination was made and the Rhinoplasty Outcome Evaluation (ROE) questionnaire was asked to patients who underwent the septorhinoplasty as an interview and satisfaction score was recorded preoperatively. All patients were operated on by a single surgeon using both open and closed approach. Written consent was taken from the patient for the procedure. Our main aim was to correct both functional and aesthetic problems related to the nose and to assess the satisfaction of patients preoperatively and postoperatively. All the cases were done under general anaesthesia. The average time taken for the procedure was around 150 minutes. After completion of the surgery, a quilting suture was done on the septum and internal nasal packing was rarely used. All the patients were admitted for 1 day and routine antibiotics were prescribed for 1 week during discharge. The sutures and external nasal splint were removed after 1 week. Routine follow up was done after 2 weeks and 4 weeks postoperative period.

Patients were studied as per their sex, ethnicity, approach and postoperative satisfaction level. The preoperative and postoperative assessment was done with the help of the ROE questionnaire.^5^ In 2000, Alsarraf^5^ developed questionnaires to assess the results of facial aesthetic surgeries that enabled the conversion of subjective patient information into quantitative data. The questionnaire was composed of 6 questions and each question has five options between 0 and 4. For all six questions, the total score was calculated and the result was divided by 24 and multiplied by 100 (Zero represents minimum satisfaction and 100 the maximum one). The questionnaire was converted into both Nepalese and English version. The questions were asked as an interview to give maximum comfort to the patients in the preoperative period. The patients were taught how to score the questionnaire in the postoperative period for data collection. This questionnaire was sent to the patient using the Viber application in 3 months, 6 months and 1 year apart and they were asked to fill the form and send it back to us. In this way, data was collected postoperatively and was calculated. Statistical Package for the Social Sciences was used for analysis.

## RESULTS

Among 5,760 patients, 117 (2.03%) underwent septorhinoplasty during the study period (Confidence Interval= 1.66-2.40). Among 117 patients, 67 (57.3%) were males and 50 (42.7%) were females with age ranging from 18-50 years were enrolled in our study.

**Figure 1.**
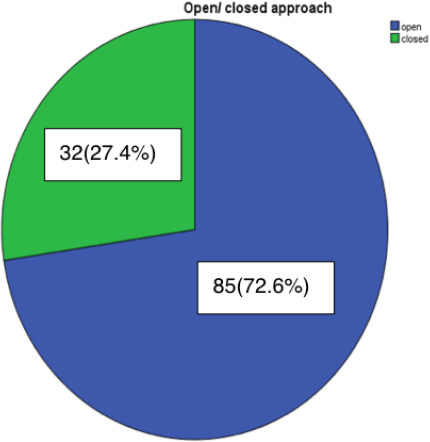
Distribution of patients undergoing open & closed approach.

Among 117 patients, 85 (72.6%) patients underwent an open approach, while 32 (27.4%) patients underwent closed approach rhinoplasty for the correction of both aesthetic and functional problems (Figure 1).

**Figure 2.**
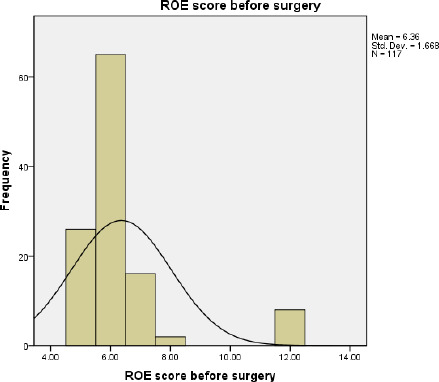
ROE score before surgery.

Different types of rhinoplasty such as reduction 14 (12%), augmentation 17 (14.5%), corrective 31 (26.5%), tiplasty 39 (33.3%), alarplasty 6 (5.1%), columelloplasty 4 (3.4%) and revision 6 (5.1%) rhinoplasty were done in these patients. The mean of ROE score before surgery is 6.36±1.668 (Figure 2).

**Figure 3.**
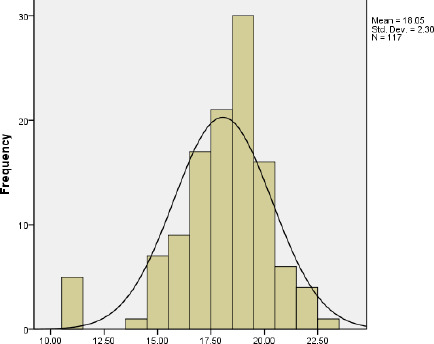
ROE score 3 months after surgery.

**Figure 4.**
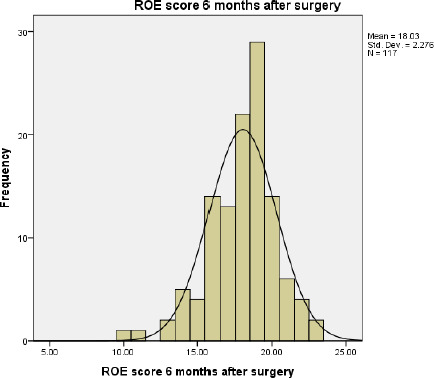
ROE score 6 months after surgery.

**Figure 5.**
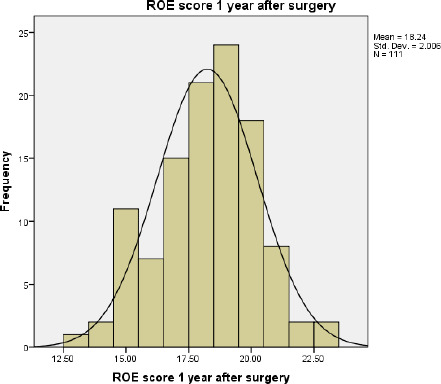
ROE score 1 year after surgery.

Likewise, the mean of postoperative ROE score after 3 months, 6 months and 1 year are 18.05±2.30, 18.03±2.276, 18.24±2.006 respectively (Figure 3, 4, 5).

## DISCUSSION

Since the nose is the central most part of the face, so any kind of nasal deformity may cause high levels of psychological distress on the patient and affect his/her quality of life. Rhinoplasty Outcome Evaluation (ROE) questionnaire created by Plastic Surgeon Alsarraf^5^ in 2000, is a screening tool for finding patient's satisfaction for the aesthetic outcome before and after septorhinoplasty. Normality values for the ROE questionnaire were studied by Izu, et al.^7^ to get better postoperative results.

In our study, we noticed that majority of patients undergoing rhinoplasty were male (57.3%) which was similar to the study done by Erdem & Ozturan.^8^ This may be explained because of correction of the deviated septum which is more common in males due to more exposure to trauma. In contrast, rhinoplasty for cosmetic purpose, female preponderance had been seen in the study done by Ferraro, et al.^9^ where males were less likely to seek rhinoplasty than females (20% males: 80% females).

Most of the patients were between 18-50 years age group with a mean age of 28 years. This age group is working and young and more concerned with the external appearance of the nose. In the preoperative, we noticed that 93.16% of patient had a satisfaction of <50. In postoperative, there was 95.73% patients in 3 months, 98.29% in 6 months and 100% in 1 year with satisfaction score above 50. A score <50 was considered as failure, a score 50 to <75 was considered to be good (32.43%) and a score >75 considered to be an excellent outcome (67.57%). Five patients in 3 months and 2 patients in 6 months had a score <50 postoperatively. But there was no worsening of satisfaction. The results were pre-counselled to patients because of thick skin. So, we noticed that 100% of patients had improvement from preoperative to postoperative satisfaction in 1-year follow-up.

In our study, the minimal pre-operative satisfaction value was 5 (20.80%) and the maximum postop satisfaction value was 23 (95.83%). The mean preoperative satisfaction level in total patients was 6.36 (26.5%), while the mean postoperative satisfaction level was 18.05 (75.20%) after 3 months, 18.03 (75.12%) after 6 months and 18.24 (76%) after 1 year of surgery. This result was similar to the study done by Biggs, et al.^10^ where the mean ROE score (postoperative) was 73.3%. Various studies done had patient postoperative satisfaction scores within the mean range of 69.75-85.4%.^11-17^

Most of the females underwent rhinoplasty for cosmetic purpose and were less satisfied than males following rhinoplasty. The mean preoperative satisfaction in male patients was 6.30 (26.25%) and in female patients was 6.44 (26.8%). The mean postoperative satisfaction in male patients was 18.29 (76.20%) and in female patients was 18.17 (75.70%). In our study men had a slightly higher level of postoperative satisfaction. These observations were similar to the study done by Tenthly Deepalakshmi, et al.^18^ Female patients were with high expectation levels and require preoperative counselling regarding their expectations. It is important to predict the results of surgery preoperatively to avoid harassment by the patient and legal actions.^19^

Many authors agree that African American patients seeking rhinoplasty desire a nose that fits their face and enhances nasofacial equilibrium, rather than changing their ethnic characteristics.^20^ This view is consistent with the results of our study, which suggest that Mongolian patients are not looking to change their ethnic features; rather, they want a nose that is in harmony with their other facial features.

## CONCLUSIONS

This study concludes the prevalence of septorhinoplasty is low which may be due to the COVID-19 pandemic during the study period. The results of the study suggest that septorhinoplasty has high satisfaction rates concerning the final aesthetic result. The grade of satisfaction remains high in both genders postoperatively. The most important factor that influences success is probably your choice of the rhinoplasty surgeon. People are pleased with the results of cosmetic nose surgery if undertaken with realistic expectations. The goal of the surgery is to create harmony and a natural shape.
